# Effect of Silica Fume on Metakaolin Geopolymers’ Sulfuric Acid Resistance

**DOI:** 10.3390/ma14185396

**Published:** 2021-09-18

**Authors:** Oliver Vogt, Neven Ukrainczyk, Eddie Koenders

**Affiliations:** Institute of Construction and Building Materials, Department of Civil and Environmental Engineering, Technical University of Darmstadt, 64287 Darmstadt, Germany; ukrainczyk@wib.tu-darmstadt.de (N.U.); koenders@wib.tu-darmstadt.de (E.K.)

**Keywords:** geopolymer, metakaolin, silica fume, sulfuric acid attack, corrosion, durability, SEM-EDX

## Abstract

To demonstrate the importance of the Si/Al ratio in terms of geopolymer mix designs for acid resistance, a metakaolin-based geopolymer was modified by replacing the aforementioned precursor with different percentages of silica fume. Durability tests were performed by exposing geopolymers with varying amounts of silica fume (up to 9%) to sulfuric acid solution (pH 1) over a period of 84 days. Geopolymer samples were analyzed by X-ray diffraction (XRD), Fourier-transform infrared spectroscopy (FTIR) and scanning electron microscopy with energy-dispersive X-ray spectroscopy (SEM-EDX) before and after 7, 14, 28, 56 and 84 days of exposure. To show the time-dependent change of the elemental composition in the corroded layer after sulfuric acid attack, SEM-EDX elemental mappings were conducted and divided into 100 µm segments to generate element-specific depth profiles. The results show that above a critical silica fume content, the erosion of the sample surface by complete dissolution can be prevented and higher amounts of silica fume lead to a significant densification of large (protective) areas of the corroded layer, which delays the progress of corrosion.

## 1. Introduction

Deterioration of concrete due to abiotic sulfuric acid attack can cause severe damage in power plant cooling towers, while biogenic conditions are met in biogas plants [1], and sewers [2], where the acid attack is microbially induced. In many countries around the world, enormous financial resources are required to maintain and repair the condition of concrete structures in such harsh environments, as no sustainable material currently exists that can withstand it in the long term [3]. 

A promising alternative to conventional cementitious binders is the use of geopolymers, which proved to have a high acid resistance in several studies [4,5] and showed better performance compared to cementitious binders [6,7,8,9,10,11]. Geopolymers are inorganic aluminosilicate polymers, which are manufactured by mixing a powdery aluminosilicate precursor with an alkaline solution or an acid to dissolve the powder precursor and initiate the subsequent processes of geopolymerization [12,13].

The aluminosilicate network of the geopolymer consists of silicon (Si) and aluminum (Al) tetrahedrons [14], cross linked by oxygen-bridging bonds [15], and charge-balancing alkali metals (Na^+^, K^+^) [16]. Since the proportion of Na^+^ resp. K^+^ in the alkaline solution usually exceeds the proportion of alkali metals required to charge balance the negatively charged Al-tetrahedrons, certain amounts of Na^+^ resp. K^+^ remain as free alkalis in the pore solution [5].

To obtain the typical aluminosilicate structure, powder precursors with relatively low proportions of calcium oxide (CaO), such as metakaolin [6,17,18,19,20,21,22] and fly ash [7,8,23,24,25,26,27,28,29], are applied. Compared to fly ash, metakaolin is more suitable, as it contains a favorable Si/Al ratio [30,31], is highly reactive in alkaline media [32] and exhibits high early and final strength when cured at room temperature [33].

Due to the lack of sufficient amounts of alumina, silica fume alone is not a suitable precursor for geopolymers. For this reason, the pozzolan is used to manufacture alkali silicate solutions [34,35,36], to increase the SiO_2_/K_2_O resp. SiO_2_/Na_2_O modulus of an existing alkali silicate solution [37] or to improve the chemical–physical properties of the geopolymer microstructure [38,39].

When geopolymers come into contact with an acidic solution, the charge-balancing alkali metals and the free alkalis in the pore solution are leached out of the system in the first step [40]. This may not necessarily lead to the destruction of the gel network, as the charge-balancing K^+^ and Na^+^ can be replaced by other cations [41,42]. The actual deterioration of the network results from the dealumination of the polymeric structure, where the aluminum is extracted due to the hydrolysis of the Si–O–Al bonds and the subsequent hydrolysis of the Si–O–Si bonds [40].

To evaluate the durability of geopolymers, in most studies, sulfuric acid [8,11,17,18,19,23,24,25,26,27,28,29,38,39,40,43,44], nitric acid [24,27], hydrochloric acid [18,20,21,22,39,45,46,47] and acetic acid [6,7,42,48,49,50] were applied. Tests with sulfuric acid may be seen as an accelerated laboratory-testing procedure compared to microbially induced sulfuric acid attack in sewers [51], as it can take several years to build up a pH of 1 on a biofilm at a sewer surface [52].

In the past, crucial insights on the acid-induced corrosion of geopolymers have been revealed. Depending on the geopolymer composition and on the type and pH of the acidic solution, the extent of corrosion will be more-or-less pronounced [8,53,54]. The outer parts of the geopolymer can erode (i.e., fully dissolve and/or detach) and a corroded surface layer can form, where the leaching of alkalis may extend beyond the corroded area, which is visually distinct from the intact core in most cases [40].

According to Lloyd et al. [27], the chemical reaction at the beginning of the degradation changes into a diffusion-controlled mechanism, which is explained by the precipitation of new reaction products inside the corroded surface layer, which act as a barrier that delays the corrosion. In most cases, the new reaction products are silicon-rich species formed by polycondensation reaction [8,9]. They comprise amorphous silica gel [8,40,46], most likely being formed in the outer areas of the corroded specimens with a pore blocking function [40], or crystalline zeolites, being either responsible for strength loss after acid attack [8], or for the densification of the corroded layer [17].

The acid resistance of the inorganic binder is also affected by the Si/Al ratio of the geopolymer, a ratio that increases over the course of acid exposure due to the dealumination of the aluminosilicate gel [8,51,55]. Higher proportions of Si-tetrahedrons connected via oxygen-bridging bonds in the initial network result in a higher polymerization degree after dealumination and, therefore, to a more stable (less soluble) gel network [40]. As a result of this, the corrosion rate can be reduced [27], which is, however, also explained by a denser matrix at higher Si/Al ratios. Furthermore, a higher Si/Al ratio can also reduce the rate of dealumination, as shown by Aly et al. [56].

In this study, silica fume was applied to gradually increase the overall silicon content of geopolymers based on metakaolin and potassium silicate solution, as the silica-rich pozzolan can increase the density of the alumosilicate network [39] and improve the acid resistance of the geopolymers [38]. Due to the already mentioned possibilities and advantages of silica fume in alkali activated binders resp. alkaline solutions, silica fume was chosen in preference to alternative silicon sources such as rice husk ash, as its high reactivity and positive effect could be proven in numerous publications.

The main objective of the present work is to elucidate the elemental composition of the corroded layer of geopolymers attacked by sulfuric acid at different durations of exposure. This was already undertaken in a comparable way by Vogt et al. [43], whereby the variation of the silicon content in the geopolymer was not taken into account and the predominant part of the corroded layer was neglected. In this context, elemental mappings performed by SEM-EDX were chosen as the main method of analysis, complemented by other methods, namely XRD, FTIR, MIP (Mercury Intrusion Porosimetry), ICP-OES (Inductively Coupled Plasma-Optical Emission Spectrometry) and mechanical strength.

## 2. Materials and Methods

### 2.1. Materials

For the synthesis of geopolymers, an industrial metakaolin (NEWCHEM, Baden bei Wien, Austria) with a density of 2.68 g/cm^3^, a specific surface area of 0.99 m^2^/g and a medium grain size of 41.4 µm was used. To increase the amount of silicon in the geopolymers, silica fume (Elkem, Oslo, Norway), with a density of 2.34 g/cm^3^ and a particle size fraction <45 µm of 98.5%, was applied. The chemical compositions of the precursors are shown in Table 1. The main components of the metakaolin were SiO_2_ and Al_2_O_3_. Other minor fractions were Fe_2_O_3_, CaO and TiO_2_, as well as Na_2_O, K_2_O and MgO with significantly lower shares. Silica fume consists mainly of SiO_2_, all other components such as Al_2_O_3_, Fe_2_O_3_, CaO, Na_2_O, K_2_O and MgO have proportions <1%.

The alkaline activation of the powdery precursors was achieved with a potassium silicate solution (Wöllner GmbH, Ludwigshafen, Germany). Relevant material properties of the product included the molar SiO_2_/K_2_O ratio of 1.5, the pH of 13.5, the density of 1.51 g/cm^3^, the viscosity of 20 mPas and the solid content of 45%. The mass fraction of SiO_2_ (22.0%) and K_2_O (23.0%) was calculated by considering the solid content and the molar SiO_2_/K_2_O ratio.

### 2.2. Geopolymer Composition

To analyze the influence of silica fume on the acid resistance of geopolymers, four different pastes were investigated, which are named GP0, GP6.0, GP7.5 and GP9.0. Sample GP0 contained only metakaolin and potassium silicate solution. For mixtures GP6.0, GP7.5 and GP9.0, the mass of metakaolin was partially replaced by silica fume. With this approach, the share of silica fume in the total mass of powder precursors (metakaolin, silica fume) amounted to 6.0% (GP6.0), 7.5% (GP7.5) and 9.0% (GP9.0). The mass of potassium silicate solution was kept constant in all of the four geopolymers, leading to an *l*/*s*-ratio (liquid/solid) of 0.54, where *l* represents the mass of the potassium silicate solution and *s* the total mass of metakaolin and silica fume. The mass fractions of precursors in the pastes are shown in Table 2. The highest metakaolin mass fraction of 64.81% (GP0) resulted from the applied *l/s*-ratio of 0.54, which enabled a good workability of the paste. Silica fume mass fractions of 3.89%, 4.86% and 5.83% resp. metakaolin mass fractions of 60.92%, 59.95% and 58.98% arose as a result of partially replacing the mass of metakaolin by silica fume (6.0%, 7.5% and 9.0%).

When comparing geopolymers before and after exposure, the designations of the geopolymers were expanded. Sample GP6.0(ref) represented GP6.0 after 28 days of curing, before exposure to sulfuric acid. GP6.0(7) designated GP6.0 after 7 days of sulfuric acid exposure. The mole fraction of each element in the pastes was calculated with the results of the chemical composition of the metakaolin and the silica fume (see Table 1), the mass fraction of SiO_2_ and K_2_O of the potassium silicate solution, the mass fraction of metakaolin, silica fume and potassium silicate solution in the geopolymer pastes (see Table 2), and the molar mass of each element. The mole fraction of the relevant elements of the geopolymers, which were identified by XRF-analysis (see Table 1), are shown in Table 3.

The H_2_O content of the potassium silicate solution was neglected. The mass fraction of elements was transformed to mole fractions to enable the comparison of the calculated mole fractions (see Table 3) with the mole fractions of the unexposed geopolymer samples, determined by SEM-EDX (see Section 4, Table 6). With the mole fractions of Si and Al, the molar Si/Al ratios of the pastes were calculated as 1.83 (GP0), 2.01 (GP6.0), 2.06 (GP7.5) and 2.11 (GP9.0). The calculated ratios typically differed from the actual Si/Al ratios of the aluminosilicate polymer, due to crystalline phases (e.g., quartz impurities), which were much less involved in the geopolymerization reaction and deviation resp. incomplete degrees of reaction.

### 2.3. Specimen Preparation

Fresh geopolymer pastes were prepared with a standard planetary mortar mixer (E092-01N Mixmatic, Matest, Arcore, Italy). In alkaline solutions, the elemental silicon in the silica fume can lead to the formation of hydrogen gas [57], a process triggered by the oxidation of elemental silicon by water (4H_2_O + Si → 2H_2_ + Si(OH)_4_) [58]. To avoid this foaming and deteriorating effect for the fresh and hardening geopolymer pastes, silica fume was premixed with potassium silicate solution 24 h before the actual mixing procedure of all precursors. After 24 h of continuous rotation of the potassium silicate solution containing silica fume, the modified alkaline solution and the metakaolin were mixed with the aforementioned planetary mortar mixer for 10 min to achieve a homogeneous paste.

For the compressive strength test, pastes were cast in polystyrene prism molds (160 mm × 40 mm × 40 mm). Specimens for the exposure to sulfuric acid were manufactured by using cylindrical polyethylene molds with a height of 35 mm and a diameter of 28 mm. Both types of molds were filled with geopolymer paste immediately after the end of mixing and compacted on a vibration table to remove air bubbles in the fresh paste.

All specimens were cured at ambient temperature (21 °C, 50% RH) up to the date of testing. To avoid loss of moisture during the course of curing, specimens in prism molds were demolded after one day of curing and wrapped in polyethylene film and aluminum adhesive tape for further curing. Specimens for exposure to sulfuric acid were kept sealed with the screw cap of the cylindrical molds. Further steps of sample preparation for sulfuric acid-exposed specimens are described in detail in Section 2.5.

For XRD and FTIR analysis, geopolymers were crushed and grinded with acetone until a fine homogeneous powder was obtained. Crushing and grinding was performed manually with an Agate mortar and pestle to minimize possible influences of the process on the reaction products [59]. In a second step, the powder was (micro-) sieved to remove all particles > 40 µm. Acetone was chosen to stop the reaction resp. to enable the rapid evaporation of water in the sample, as this procedure was proven to be affective in previous studies [33,60,61].

### 2.4. Exposure to Sulfuric Acid

**Figure 1:**
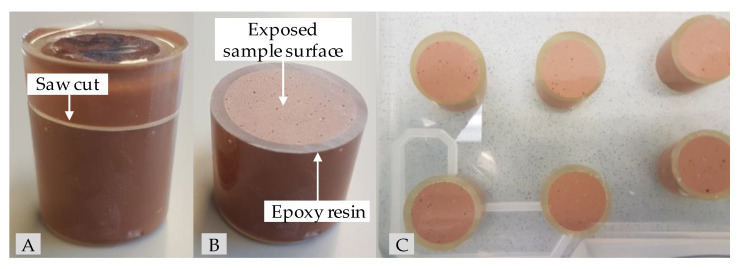
Epoxy-embedded geopolymer specimen for sulfuric acid exposure before (**A**) and after saw cut (**B**) as well as geopolymers GP7.5 ((**C**), top row) and GP9.0 ((**C**), lower row) after one day in sulfuric acid (pH 1).

As biogenic conditions can result in a pH of 0 [62], but in most cases the pH is in the range 1–3 [63], concentrated sulfuric acid (96%) and deionized water were used to prepare a sulfuric acid solution with a pH of 1 to represent a situation as close to the real field application as possible. To achieve a one-dimensional diffusion-controlled acid attack, the specimens for the exposure were completely coated in epoxy resin after 26 days of sealed curing. After two more days, when the geopolymer pastes had reached an age of 28 days, the epoxy-coated specimens were saw cut orthogonally to the height of the specimen (see Figure 1A). Due to the partially coated specimen surface (see Figure 1B), the exposed surface of a specimen could be reduced to an area of 6.16 cm^2^. The ratio *V*_A_/*A*_S_, where *V*_A_ represents the volume of sulfuric acid (dm^3^) and *A*_S_ the total surface of the specimens exposed to the acid (dm^2^), was set to 9.7. Immediately after the saw cutting, the specimens were inserted into the sulfuric acid solution and remained at its position until the date of testing (see Figure 1C).

To avoid deviations in the properties of the sulfuric acid over the course of storage, the specimens of all four geopolymers were stored in the same tank. Over the whole period of 84 days of exposure, the solution was not changed. The pH of the solution was measured each day with a pH meter (Hanna pH 211, Vöhringen, Germany), as an average of two replicate setups, and kept constant at approximately pH 1 by adding a 50% concentrated sulfuric acid solution, similarly to the approach of Vogt et al. [43]. The solution was carefully stirred manually before the daily pH measurement. An automatic continuous stirring was not applied, since an acceleration of the degradation process should be avoided [53]. The corroded samples were analyzed after 7, 14, 28, 56 and 84 days of exposure with the appropriate methods (see Section 2.6).

### 2.5. Sample Preparation after Exposure

At the date of testing, the specimens for SEM-EDX analysis were carefully removed from the solution and dried at 40 °C in a drying oven (UT6P Heraeus, ThermoFisher, Waltham, MA, USA) until mass constancy. Orthogonally to the exposed surface of the specimen, 1 cm thick sections with a length of 2 cm were dry cut with a low-speed diamond-tipped precision cutter (IsoMetTM, Buehler, Esslingen am Neckar, Germany). The cross-sections were impregnated with a low viscosity epoxy resin (Epofix, Struers, Cleveland, OH, USA), which had been cured at 40 °C for 24 h before polishing with a polishing machine (Labo-Force 100, Struers, Cleveland, OH, USA). Polishing was performed with a resin-bonded diamond disc (hardness range: HV 150 to 2000) at a rotational speed of 300 rpm to reveal the epoxy-coated surface of the specimen. For the final polishing, an automated polycrystalline diamond spray was applied at a rotational speed of 150 rpm.

### 2.6. General Methods

Compressive strength tests of specimens after 1, 7 and 28 days of sealed curing were performed with half prisms (80 mm × 40 mm × 40 mm) according to DIN EN196-1 [64] (load increase 2.4 kN/s). Each value of the compressive strength was the mean value of six individual measurements.

Mercury intrusion porosimetry (MIP) analysis was conducted with a Pascal 440 Mercury Porosimeter (ThermoFisher, Waltham, MA, USA). The stopping of reaction and the removal of water from the specimens before MIP analysis was achieved by immersing the samples in liquid nitrogen and subsequent storage in a freeze dryer (Lyotrap, LTE Scientific Ltd., Oldham, UK) until the mass change of each specimen changed by no more than 0.2% within 24 h [65]. Relevant parameters for the measurements included the contact angle (125°) and the surface tension (0.48 N/m). Prior to MIP analysis, the skeleton density of the powder samples was measured with a Helium pycnometer (Pyknomatic-ATC, ThermoFisher, Waltham, MA, USA). To improve the quality of the obtained data, a mean value was calculated from three individual measurements.

The concentration of the silicon, aluminum and potassium elements in the sulfuric acid solution after 28, 56 and 84 days of exposure was analyzed by inductively coupled plasma emission spectroscopy (ICP-OES) with an Optima 2000 DV (PerkinElmer, Waltham, MA, USA). Each element concentration corresponded to the mean value from two individual measurements.

A D2 Phaser (Bruker, Hamburg, Germany) was used to perform qualitative powder X-ray diffraction of precursors and geopolymers before and after the exposure to sulfuric acid. Analysis was conducted with a CuKα_1,2_ anode as a radiation source at 30 kV and 10 mA, with an average wavelength of 0.15406 nm. Further parameters included the goniometer measurement circle of 283 mm, the primary optics with a 0.4 mm fixed slit and the secondary optics with a Ni-filter and a 2.5° soller slit. Samples were measured between 3 and 70° 2θ, with 0.02° step size and a measurement time of 2 s/step. For the qualitative evaluation of the XRD spectra, the DIFFRAC.EVA software (Bruker, Hamburg, Germany) was used. Quantitative X-ray powder diffraction of metakaolin and silica fume was achieved by applying Rietveld refinement with the DIFFRAC.TOPAS software (Version 5, Bruker, Hamburg, Germany), with corundum as an internal standard (10% spiked samples). The Rietveld quantification of amorphous phases in powder precursors was previously conducted in the literature, by considering the broad humps in the spectra [33].

Infrared Spectroscopy was performed with the ATR-FTIR-spectrometer Spectrum One (PerkinElmer, Waltham, MA, USA). The spectral range of the measurements was between 650 and 4000 cm^−1^, with a resolution of 4 cm^−1^. Small amounts of powder samples were placed on the ATR diamond crystal and pressed to its surface using the ATR-FTRI device until a transmission of the preliminary spectrum of approximately 50% was reached. For each powder sample, eight scans were conducted. The result of each measurement represented the accumulation of the eight individual scans.

SEM-EDX was performed with a Zeiss EVO LS25 SEM (Jena, Germany) and an EDX detector (EDAX, Ametek, Berwyn, PA, USA). Elemental mappings were performed in low vacuum mode (10 Pa) with an accelerating voltage of 15 keV and beam current of 2.0 nA. For each pixel of the mapping (512 × 400 pixels in total) with an image size of 3.0 × 2.4 mm, a dwell time of 500 µs and a repetition of 128 frames was chosen. ZAF correction was applied to minimize influences from the interaction between the sample surface and the electron beam. The elemental errors of each mapping were achieved by using an internal device standard, which, in contrast to the use of sample-specific standards, led to the results being classified as semi-quantitative [66].

The depth of erosion, representing the change of height of the exposed geopolymer specimen surface before and after exposure, was measured with a digital caliper gauge, starting from the epoxy resin edge of the exposed sample surface.

### 2.7. SEM-EDX Mappings

**Figure 2:**
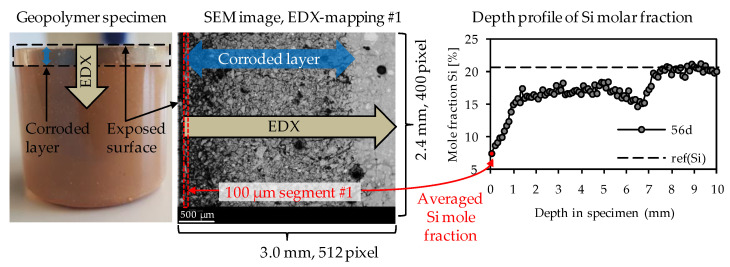
Direction of EDX-mappings, position of 1st 100 µm segment of EDX-mapping and depth profile of EDX mole fractions, exemplified for silicon.

Elemental mappings were performed as presented in Figure 2. For each specimen after sulfuric acid exposure, the elemental mappings included the corroded layer on the surface of the specimen and a certain part of the optically non-corroded area. As the corrosion progressed continuously, several individual elemental mappings were required to reach the unaltered core of the specimen. The correct transition of one mapping to the next could be ensured by the SEM-EDX images resp. the large number of characteristic pixels such as air voids or quartz grains. In order to generate the elemental depth profiles of the geopolymers (see Figure 2), each elemental mapping was subdivided into segments with an image width of 100 µm, where the width of 100 µm is orthogonal to the exposed surface of the sample. For each of those 100 µm segments, the particular elemental composition was averaged by the EDX software. The first 100 µm segment was positioned at the outermost edge of the corroded surface layer and, thus, also represented the first data point of the depth profile (see Figure 2).

The dashed horizontal line in each depth profile represented the elemental mole fraction of the geopolymer after 28 days of ambient curing before exposure to sulfuric acid, designated as ref(E), shown in Figure 2 as an example for silicon with ref(Si). By this, the sulfuric acid-induced elemental loss at any depth of the specimen could be visualized, as well as the depth where the depth profile reached ref(E). The intersection between the depth profile and ref(E) could be interpreted as the position in the specimen above which the sulfuric acid no longer caused any elemental change in the sample.

To obtain the elemental mole fractions of ref(E), geopolymer samples were analyzed after 28 days of ambient curing by SEM-EDX. Each ref(E) value is the mean value of three individual elemental mappings of the geopolymers cross-section.

For each elemental mapping of corroded samples and geopolymer samples before exposure to sulfuric acid, the following elements were taken into account: silicon (Si), aluminum (Al), iron (Fe), calcium (Ca), titanium (Ti), sodium (Na), potassium (K), magnesium (Mg), oxygen (O) and Carbon (C). The selection of elements was a result of the elemental composition of metakaolin, silica fume and potassium silicate solution. Carbon was taken into account, as the epoxy resin for SEM-EDX sample preparation contained large proportions of this element. For each 100 µm segment, the sum of the mole fractions of the above mentioned 10 elements was always 100%.

**Figure 3:**
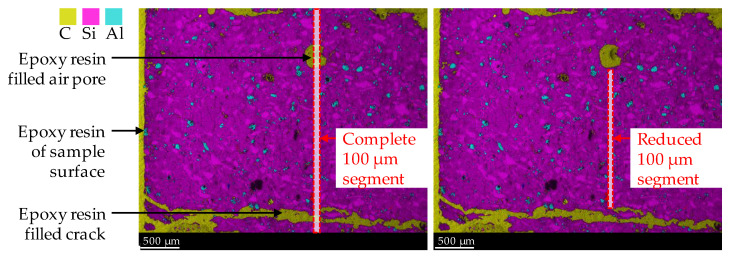
SEM-EDX mapping image of corroded geopolymer specimen. Illustration of complete 100 µm segment (including epoxy resin-filled air pores and cracks) and reduced 100 µm segment, to avoid the influence of epoxy resin and cracks on the mole fractions of elements.

By considering carbon, all epoxy resin-filled air voids and cracks in the specimen’s cross-section were detected as carbon. In addition, carbon was used for semi-quantitative analysis of the increased porosity of the geopolymer in the corroded layer due to sulfuric acid-induced corrosion, since the carbon mole fraction would increase at higher porosity, as it filled the pores during sample preparation (after the corrosion tests). Manual corrections were made to the 100 µm segments, as shown in Figure 3, to exclude epoxy resin-filled air pores and large cracks, which were, thus, not representative of the porosity of the corroded layer. The segment width of 100 µm and the image height of the elemental mapping of 2.4 mm resulted in an area of 0.24 mm^2^ resp. 6827 pixels for a complete 100 µm segment (see left image in Figure 3). During manual correction, the image height for all segments was not less than 1.2 mm. By doing so, each reduced 100 µm segment (see right image in Figure 3) had an area of at least 0.12 mm^2^ resp. 3414 pixels.

## 3. Results

### 3.1. Metakaolin and Silica Fume

**Figure 4:**
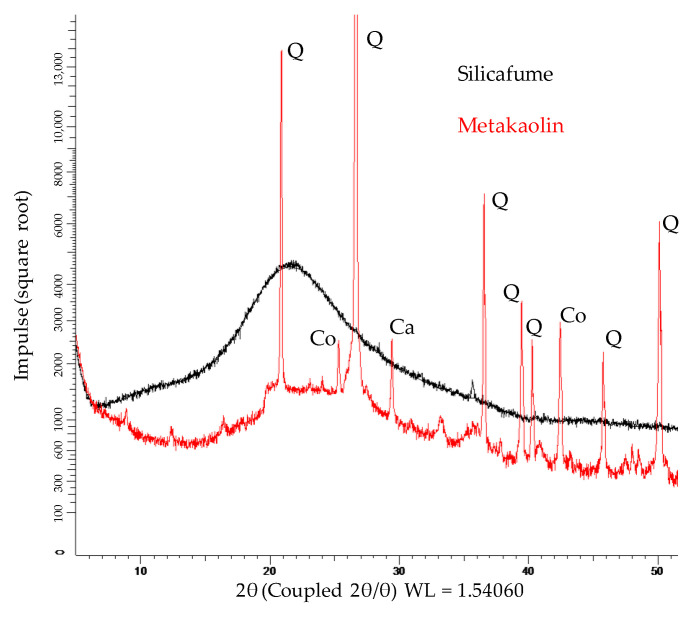
XRD spectra of metakaolin and silica fume (Q: quartz; Co: Corundum; Ca: Calcit).

**Figure 5:**
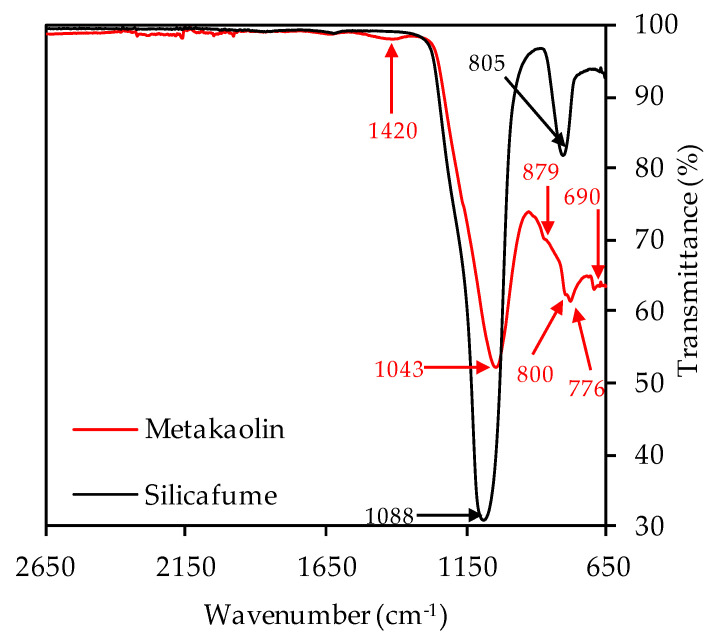
FTIR spectra of metakaolin and silica fume.

The XRD spectra of metakaolin and silica fume are shown in Figure 4. Due to Rietveld quantification, the crystalline phases and the total amount of amorphous share could be determined. The metakaolin contained 51.7% amorphous phases, 38.8% quartz and other crystalline phases such as muscovite, calcite, vaterite, sanidine, halloysite, mullite, hematite, anatase, diaoyudaoite and cristobalite in significantly smaller amounts. The silica fume consisted almost entirely of amorphous phases (98.9%), with traces of quartz, cristobalite and crystalline SiO_2_. FTIR spectra of metakaolin and silica fume are presented in Figure 5.

The strong bands at 1043 cm^−1^ (metakaolin) resp. 1088 cm^−1^ (silica fume) represent the Si–O–Si and Si–O–Al bonds [67,68]. The second strong band of the silica fume spectra at 805 cm^−1^ also represents Si-O-Si bonds of the pozzolan [69,70,71]. For metakaolin, the band at 1420 cm^−1^ can be associated with stretching vibrations of C–O–C bonds resp. carbonates [46,72,73]. These were also detected by XRD analysis, in the mineralogical formation of calcite and vaterite. The overlapping bands at 879, 800, 776 and 690 cm^−1^ are more difficult to assign to a specific bond type to, and are discussed in the scope of the FTIR spectra of unexposed and exposed geopolymers (see Section 3.2.3 and Section 3.4.3).

### 3.2. Unexposed Specimens

#### 3.2.1. Compressive Strength

Table 4 shows the compressive strength of the geopolymers after 1, 7 and 28 days of sealed curing at ambient temperature. For GP7.5 and GP9.0, the strength after 1 resp. 7 days of curing was too low to detect with the testing device. For GP0, the highest strength was achieved after only one day of curing, with lower strength after 7 and 28 days, although the strengths after 1 and 28 days were of comparable magnitude. The evolution of the compressive strength of GP6.0, GP7.5 and GP9.0 illustrates that strength was strongly influenced by the addition of silica fume, which is discussed in more detail in Section 4.

#### 3.2.2. Mercury Intrusion Porosimetry

**Figure 6:**
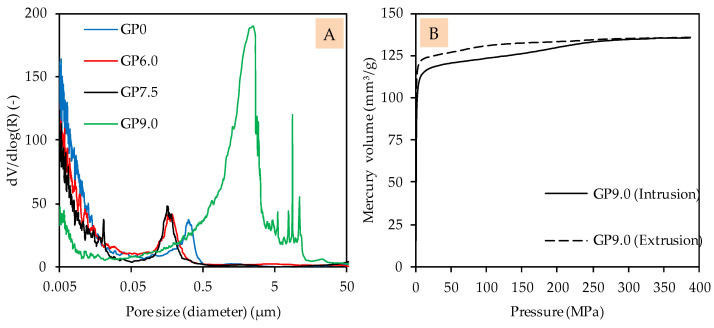
Pore size distribution of geopolymers after 28 days of curing (**A**) and pressure-dependent mercury volume in sample for intrusion and extrusion of GP9.0 after 28 days of curing (**B**).

The pore size distribution of GP0, GP6.0 and GP7.5 indicates roughly similar pore structures, with only a slight shift in pore sizes towards smaller pores, as a result of the addition of silica fume (see Figure 6A). In the case of GP6.0 and GP7.5, the shift of pore size distribution towards smaller pores may be due to the denser matrix of silica fume-modified geopolymers, in agreement with the improved compressive strength. Compared to this, the pore size distribution of GP9.0 shows a completely different picture, which may be explained by the lower reactivity and/or fragile pore structure, which may collapse during the MIP test (see Figure 6B).

The intrusion curve indicates that the geopolymer may have become destroyed over the course of the test, since relatively high amounts of mercury were intruded without an increase in device pressure. This result confirms the observation made in the context of the evolution of the compressive strength, since GP9.0 does not appear to have formed a sufficiently strong network, after 28 days of curing, to withstand MIP analysis.

#### 3.2.3. Fourier-Transform Infrared Spectroscopy

**Figure 7:**
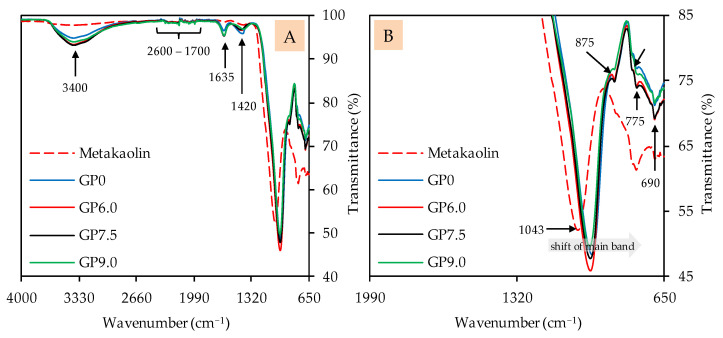
FTIR spectra of metakaolin and geopolymers after 28 days of curing. Full (**A**) and partial spectra (**B**).

The FTIR spectra of geopolymers after 28 days of ambient curing, before exposure to sulfuric acid, are presented in Figure 7 (image A: complete spectra; image B: partial spectra). The metakaolin spectra are also included to compare certain bands of the precursor with the bands of the geopolymers. The broad band, with its peak at approx. 3400 cm^−1^, comprises O–H stretching vibrations [67,69,72] or indicates the absorption of water molecules [69,70]. The multiple weak bands in the range between 2600 cm^−1^ and 1700 cm^−1^, which can be seen in the metakaolin spectra as well as in the geopolymer spectra, indicate unreacted components of metakaolin [17]. The bands at approx. 1635 cm^−1^ can be associated with bending vibrations of O–H and absorbed water molecules [67,69,70], which, comparably to the bands at 3400 cm^−1^, are more pronounced for the geopolymers than for metakaolin. The stretching vibrations of C–O–C bonds resp. carbonates [46,72,73], located at approx. 1420 cm^−1^, are stronger in the geopolymer spectra as compared to the metakaolin spectra. This is probably due to contact with air during sample preparation (drying, grinding), since K^+^, in particular, can react with atmospheric CO_2_ to form K_2_CO_3_.

The shift of the strong main band of the metakaolin spectra (1043 cm^−1^), representing Si–O–Si and Si–O–Al bonds [67,68], towards lower wavenumbers is an indication of the aluminosilicate gel formation of the amorphous geopolymer [74]. The Si–OH bonds at approx. 875 cm^−1^ [68,75], which are less pronounced in geopolymers compared to metakaolin, are not involved in the geopolymer gel formation [76]. For the bands at 795 cm^−1^, 775 cm^−1^ and 690 cm^−1^, the literature specifies different types of bonds. The band at 795 cm^−1^ could be Si–O–Si bonds [69,70] or Al–O bonds [77]. The band at 775 cm^−1^ could be Al–OH and Al–O bonds [74,78], but also Si–O–Si and Si-O-Al bonds [78]. For the band at 690 cm^−1^, quartz [73], but also Si–O–Si resp. Si–O–Al bonds [17,22,73], were named in the literature.

### 3.3. Sulfuric Acid

**Figure 8:**
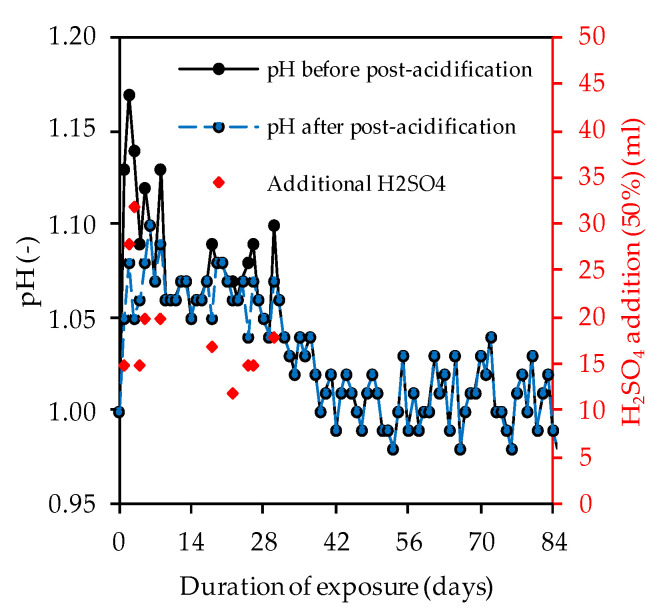
pH of sulfuric acid solution over the course of exposure and volume of additional H_2_SO_4_.

Figure 8 clearly shows that the pH of the acidic solution increased sharply during almost every day of exposure, whereby the neutralization of the sulfuric acid occurred as a result of the ion exchange between the acid and the alkaline sample [48]. Within the first 28 days of exposure, varying amounts of concentrated sulfuric acid were required to keep the pH more-or-less constant at pH 1.

**Figure 9:**
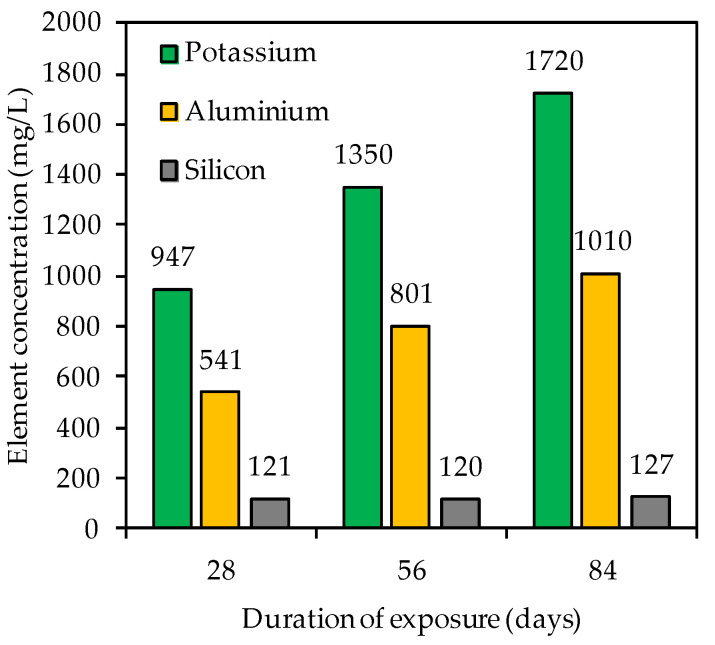
Potassium, silicon and aluminum concentration in sulfuric acid solution after 28, 56 and 84 days of exposure.

Figure 9 contains the element concentration (mg/L) of potassium, aluminum and silicon in the acidic solution after 28, 56 and 84 days of exposure. For each testing date, the potassium concentration exceeded the aluminum concentration and the silicon concentration was always lower than the aluminum concentration. These changes in elemental concentrations are in agreement with other studies [6,56,79]. Aly et al. [79] assumed that Si is most strongly embedded in the geopolymer. However, the authors also indicated that dissolved Si and Al may react with OH^−^ to form Si(OH)_4_ and Al(OH)_3_. Moreover, the total element concentration determined by ICP-OES (Figure 9) does not necessarily correspond to the concentration dissolved from the specimen, due to possible precipitation from oversaturated solution [44].

For potassium and aluminum, a continuous increase in concentrations could be observed (Figure 9) over the course of exposure, whereas the silicon concentration did not change significantly. The differences in element concentrations are consistent with the statements on the corrosion mechanism of geopolymers; specifically, in the first step of the acid attack, more alkalis are leached out, followed by the partial dealumination of the alumosilicate network, whereas the last step is typically characterized by a higher release of Al concentrations [48].

### 3.4. Corroded Specimens after Sulfuric Acid Exposure

#### 3.4.1. Visual Analysis

**Figure 10:**
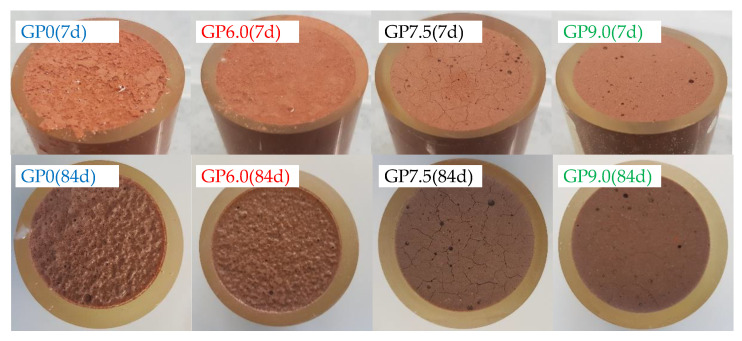
Geopolymer specimens immediately after removal from sulfuric acid, after 7 and 84 days of exposure.

The visual analysis of geopolymer specimens after 7 and 84 days of exposure, immediately after removal from the sulfuric acid, illustrates the influence of silica fume addition in the geopolymer formulations (see Figure 10). After only seven days of exposure, GP0 and GP6.0 showed a partially dissolved layer and sediments of dissolved particles on the specimen’s surface. In contrast, no dissolution of solid material was observed in samples GP7.5 and GP9.0, even after 84 days of exposure. However, the observed surface cracks on GP7.5 and GP9.0 suggest that a reaction nevertheless occurred in the specimens containing higher proportions of silica fume.

In the present study, surface cracks were first noticed after seven days of exposure (GP7.5) resp. 28 days of exposure (GP9.0). An explanation of the different cracking behaviors might be the varying strength of the geopolymers and also the constraining stresses caused by the epoxy resin surrounding the specimen. The formation of new minerals such as alunite, K-567 alum, syngenite and anhydrite, as detected by Grengg et al. [44] after exposure of metakaolin-based geopolymers in sulfuric acid, could also be one possible reason for at least some of the cracks.

#### 3.4.2. Depth of Erosion

The depth of erosion, listed in Table 5, is in agreement with what has already been described in the previous section. Higher silica fume proportions in the formulations, GP7.5 and GP9.0 did not exhibit a depth of erosion, since the surface before and after exposure did not record a change in height. Comparing GP0 to GP6.0, GP0 had a slightly greater depth of erosion at all testing dates. The evolution of depth of erosion over the complete period of acid exposure did not show a clear trend, suggesting that the process of erosion is more-or-less completed after seven days of exposure.

#### 3.4.3. Fourier-Transform Infrared Spectroscopy

**Figure 11:**
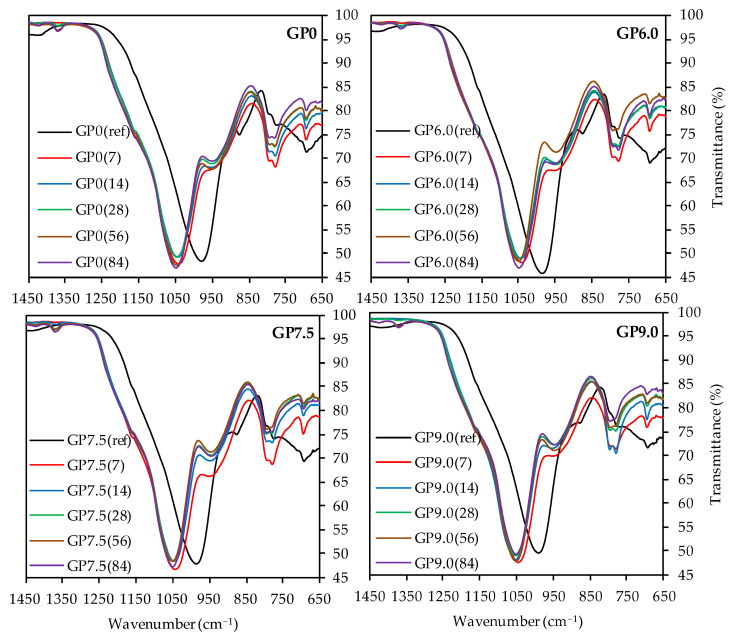
FTIR-spectra of geopolymers GP0, GP6.0, GP7.5 and GP9.0 before (ref) and after exposure to sulfuric acid (7, 14, 28, 56, 84).

As the depolymerization of the geopolymer is the main objective of the present work and the bands at higher wavenumbers do not provide any decisive information, Figure 11 only shows the partial FTIR spectra in the range between 1450 cm^−1^ and 650 cm^−1^ of geopolymers after acid exposure, compared to the spectra before sulfuric acid attack.

For all geopolymers, the C–O–C bonds at approx. 1420 cm^−1^ [46,72,73] were hardly visible after 7 and 14 days of exposure, probably due to the dissolution of carbonates in the sulfuric acid at the beginning of acid exposure [22].

The reappearance of those bands at later testing dates might have resulted from atmospheric CO_2_ in the acidic solution, absorbed on the sample surface or within the pore structure of the specimen. The subsequent reaction could have been the formation of HCO_3_^−^ (bicarbonate) and CO_3_^2-^ (carbonate ion). As a consequence, the potassium and the carbonate ion could have formed K_2_CO_3_ (potassium carbonate) and KHCO_3_ (potassium bicarbonate) [21].

The bands at app. 1170 cm^−1^, which were hard to detect due to the overlap with the main band of the spectra, represent the quartz of the metakaolin [73,80]. The more pronounced bands of exposed samples, compared to the quartz bands of geopolymers before exposure, indicated higher proportions of quartz after sulfuric acid attack. Assuming that the acid destroyed the amorphous alumosilicate network and the crystalline quartz remained undissolved in the corroded layer of the sample, the higher proportions of quartz resulted from the reduction in the amorphous components of the geopolymer.

The main band of each spectra, which represented the Si–O–Si and Si–O–Al bonds of the geopolymer [67,68], showed a significant shift towards higher wavenumbers after exposure to sulfuric acid, an effect that has previously been observed in the literature [8,22,29]. Moreover, it is known that higher wavenumbers of the main band of geopolymers are reached with higher proportions of Si in the geopolymer gel [76], resp. an increase in the Si/Al ratio [8,81]. This leads to the conclusion that higher proportions of aluminum are dissolved from the geopolymer gel, as compared to silicon, and the corroded layer contains higher proportions of silicon after sulfuric acid exposure [46]. This would confirm the assumption that the dealumination of the geopolymer network is the main deterioration mechanism of the acid-induced corrosion. For all geopolymers, the peak of the main band before exposure is located at wavenumber range between 980 cm^−1^ and 991 cm^−1^. After sulfuric acid exposure, this range shifts to wavenumbers between 1041 and 1054 cm^−1^, with slightly higher wavenumbers for longer exposure times. This may be due to the progressing dealumination [46], although the main shift of the band already occurs after 7 days of exposure, which is an indication for the immediate dealumination of the corroded layer and only minor further changes for later exposure times [29].

The band at approx. 875 cm^−1^ in the spectra before exposure, which represents the Si–OH groups of the geopolymer [68,75], also shifts towards higher wavenumbers due to sulfuric acid exposure. In addition, lower transmittance after exposure can be observed. Despite the qualitative nature of the results, this could indicate higher proportions of Si–OH groups in the sample [8]. As the Si–OH groups do not represent the characteristic oxygen-bridging bonds between the Si and Al tetrahedrons [76], the presence of higher proportions of Si–OH is in agreement with a destruction of the amorphous network.

Although the assignment of bands at 795 cm^−1^ and 775 cm^−1^ to specific bonds is a difficult task, their wavenumbers and transmittance values of the spectra before and after exposure differ only slightly from each other. For the band at approx. 690 cm^−1^, no significant shift, after exposure to sulfuric acid, can be detected, while higher transmittance values suggest lower proportions of the corresponding bonds in the samples. In this context, Hajimohammadi et al. [76] assigned Al–O bonds at approx. 700 cm^−1^ to these bands, whereas Riyap et al. [68] clearly detected Si–O–Al bonds at 728 cm^−1^. Assuming aluminum bonds for the bands in this range, the increase in transmittance values after sulfuric acid exposure indicates the aforementioned dealumination of the geopolymer structure.

#### 3.4.4. X-ray Diffraction

**Figure 12:**
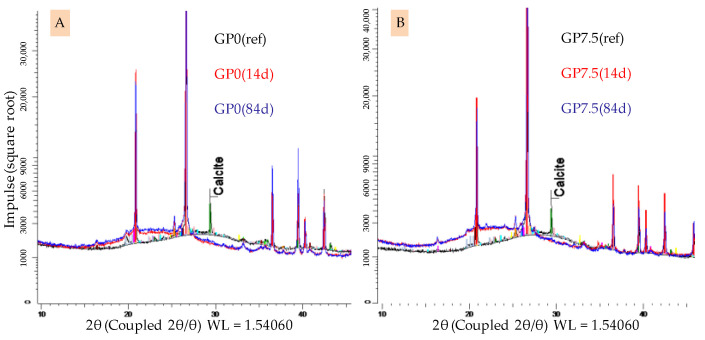
XRD spectra of geopolymers GP0 (**A**) and GP7.5 (**B**). Geopolymers before exposure to sulfuric acid (GP0(ref), GP7.5(ref)) and corroded layer after 14 days (GP0(14), GP7.5(14)) and 84 days of exposure (GP0(84), GP7.5(84)).

The XRD spectra of GP0 and GP7.5 after 28 days of curing before and after 14 and 84 days of exposure to sulfuric acid are shown in Figure 12. For both geopolymers, the typical broad hump in the spectra of geopolymers in the range of 20–35° 2θ, representing the amorphous aluminosilicate gel [47,48], shifts towards lower angles 2θ after sulfuric acid exposure.

A broad hump still being present after acid exposure with the aforementioned shift was previously reported in the literature [48,49]. Differences in the intensity of the shifts, depending on the amount of silica fume in the geopolymers, cannot be detected. The calcit peak in the spectra of geopolymers before exposure vanishes after sulfuric acid exposure. In this context, Gu et al. [51] already indicated that the complete dissolution of calcit in the geopolymer is possible due to an acid attack on the binder. The formation of new minerals, which may lead to expansion and partial destruction within the specimen, which has been reported in literature before [26,54], could not be detected.

#### 3.4.5. SEM-EDX

**Figure 13:**
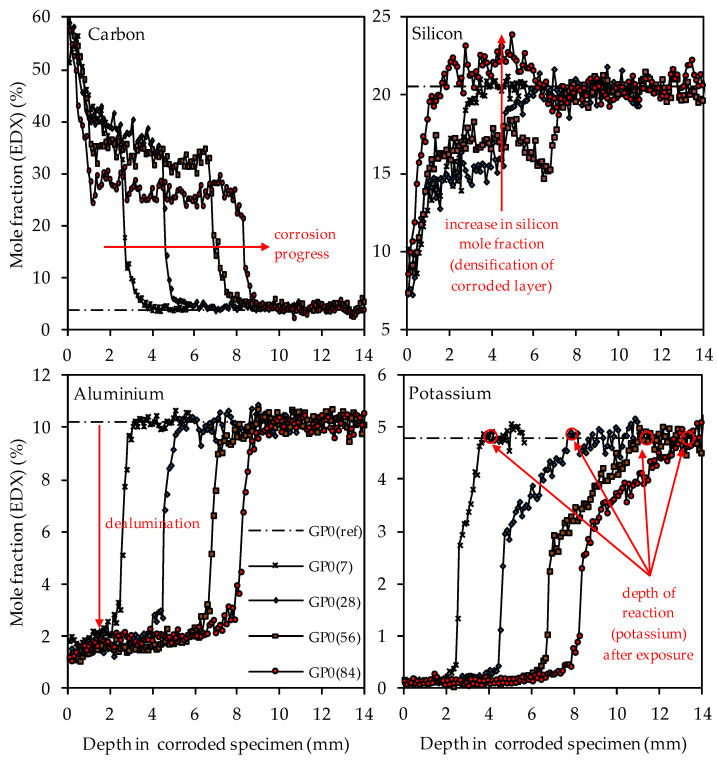
SEM-EDX mole fractions of carbon, silicon, aluminum and potassium of geopolymer GP0. Mole fractions before (ref) and after (7, 28, 56, 84) exposure to sulfuric acid. Exemplary marking of corrosion progress (carbon), densification of corroded layer (silicon), dealumination (aluminum) and depth of reaction after exposure (potassium).

Figure 13 shows the GP0 depth profiles of carbon, silicon, aluminum and potassium after 7, 28, 56 and 84 days of exposure (GP0(7), GP0(28), GP0(56) and GP0(84)) as well as the elemental mole fraction of the geopolymer after 28 days of ambient curing before exposure to sulfuric acid (GP0(ref)). For a clearer presentation of the results, the depth profiles after 14 days of exposure were omitted from all graphs, as well as the error bars for each point, i.e., the 100 µm segment.

The depth profiles of carbon, representing the higher porosity of the geopolymer matrix in the corroded layer due to epoxy resin-filled pores and smaller cracks (air voids, large cracks and larger pores were excluded, see Figure 3), clearly shows the progress of the corrosion, as the significantly higher carbon mole fractions in the corroded layer penetrate into deeper areas of the geopolymer specimens. The comparison of the depth profile after 7 days and 84 days of exposure indicates that the carbon mole fraction in the corroded layer decreased over the course of sulfuric acid exposure. For GP0(7), the carbon mole fraction in the corroded layer had an average value of approximately 40%, whereas, after 84 days of exposure (GP0(84)), the mean carbon mole fraction was in the range between 30% and 25%.

This effect can also be seen in a comparable way in the silicon depth profiles, as the silicon mole fractions in the corroded layer increased with progressive corrosion. After 28 days of exposure (GP0(28)), the mean silicon mole fraction of the corroded layer was still in the range between 13% and 16%, before the steep rise in the depth profile approached GP0(ref). After 56 days of exposure (GP0(56)), the average silicon mole fraction had reached approximately 17%. The decisive change within the corroded layer became apparent after 84 days of exposure (GP0(84)), as the silicon depth profile exceeded the silicon mole fraction of the reference geopolymer (before exposure to sulfuric acid, GP(ref)). This elemental change in large areas of the corroded layer proved what had already been stated in the literature [54], namely that the formation of new silicon-rich species may act beneficially by the subsequent densification of the corroded layer over time. The increase in the silicon mole fractions in the corroded layer also explains the decrease in carbon mole fractions in the aforementioned area, since the cavities (pores, cracks) within the geopolymer structures caused by sulfuric acid corrosion were partially filled again by new silicon species over time. Therefore, smaller areas of the specimen cross section were filled with epoxy resin over the course of sample preparation, which automatically resulted in lower carbon mole fractions.

The aluminum depth profiles demonstrate that the dealumination of the corroded layer, as the mole fraction of 10.2% (aluminum mole fraction before exposure to sulfuric acid GP0(ref)) decreased due to the sulfuric acid attack and reached values in the range between approximately 1% and 2%. No significant deviations or even an increase in the values over the course of acid exposure can be observed, as was the case with silicon. The potassium depth profiles show that the depth of reaction also progressed into the depth of the geopolymer specimen. The depth of reaction was calculated for individual elements as the point where the depth profile intersected the horizontal line in the graphs (elemental mole fraction after 28 days of ambient curing before exposure to sulfuric acid) [43]. Comparing the depth profiles of aluminum and silicon with the potassium depth profiles demonstrates that the elemental loss of potassium progressed into deeper areas of the test specimen. Due to the interaction between the sulfuric acid and the geopolymer resp. its pore solution, potassium leached out of the matrix, which led to lower potassium mole fractions in the EDX analysis. Similar to the aluminum depth profiles, no significant deviations between the potassium mole fractions in the corroded layer can be observed.

**Figure 14:**
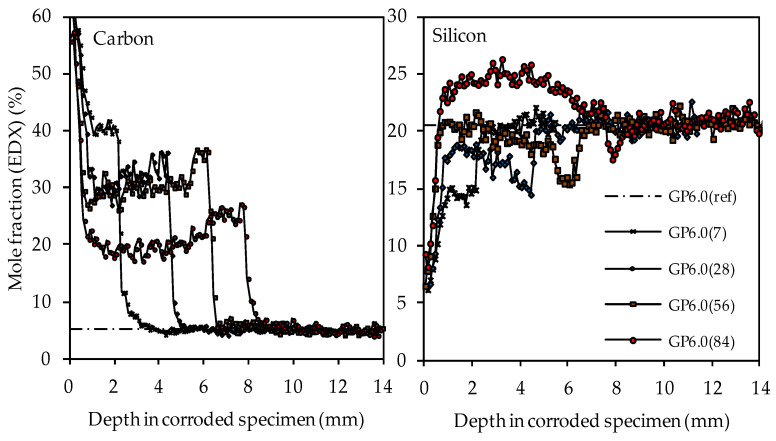
SEM-EDX mole fractions of carbon and silicon of geopolymer GP6.0. Mole fractions before (ref) and after (7, 28, 56, 84) exposure to sulfuric acid.

**Figure 15:**
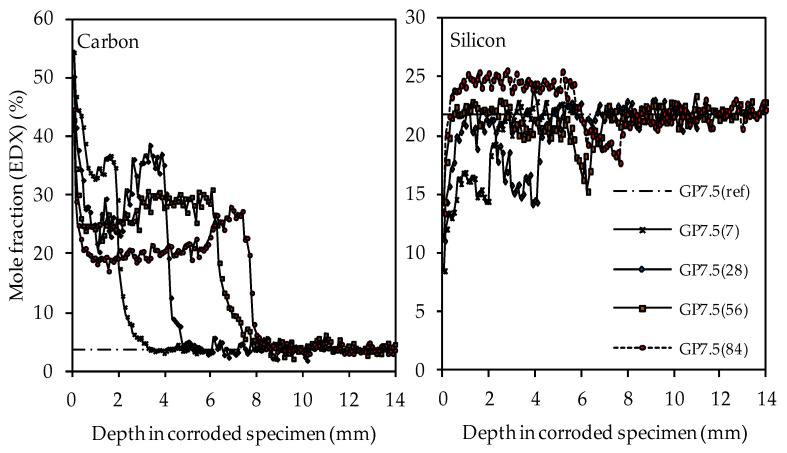
SEM-EDX mole fractions of carbon and silicon of geopolymer GP7.5. Mole fractions before (ref) and after (7, 28, 56, 84) exposure to sulfuric acid.

**Figure 16:**
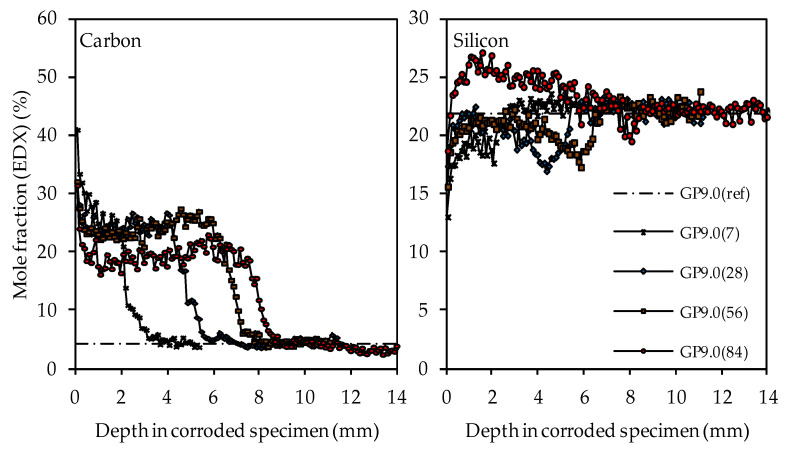
SEM-EDX mole fractions of carbon and silicon of geopolymer GP9.0. Mole fractions before (ref) and after (7, 28, 56, 84) exposure to sulfuric acid.

The carbon and silicon depth profiles of geopolymers containing silica fume are presented in Figure 14 (GP6.0), Figure 15 (GP7.5) and Figure 16 (GP9.0). The aluminum and potassium depth profiles are not displayed, as they show similar trends as those already described for GP0 (see Figure 13). Comparing the carbon and silicon depth profiles of GP6.0, GP7.5 and GP9.0 with the carbon and silicon depth profile of GP0 indicates that certain amounts of silica fume led to a more acid-resistant geopolymer network and a more pronounced densification of the corroded layer. This effect becomes most obvious when comparing the depth profiles of GP0 and GP9.0. The carbon mole fractions before exposure had only slightly different values, namely 4.0% for GP0(ref) and 4.2% for GP9.0(ref). Nevertheless, after sulfuric acid exposure, the carbon mole fractions of GP0 at the outer edge of the corroded layer reached values in the range between 50% and 60% (see Figure 13), whereas the carbon mole fractions of GP9.0 did not exceed 40% at the outer edge of the specimen (see Figure 16). Furthermore, the mean carbon mole fraction of the corroded layer was significantly lower in the case of GP9.0, for all exposure times. In the case of silicon profiles, higher amounts of silica fume led to a less pronounced loss of silicon at the beginning of exposure and a more pronounced densification in the corroded layer, especially after 84 days of exposure. Furthermore, after 56 days of exposure the corroded layer of geopolymers containing silica fume already possessed silicon mole fractions that were higher than the mole fractions before the attack, whereas, in the case of GP0, this happened only after 84 days of exposure.

## 4. Discussion

The addition of optimal amounts of silica fume to metakaolin-based geopolymers can improve the acid resistance of the inorganic binders. This could be shown most clearly by the results of the visual analysis resp. the depth of erosion and the depth profiles of the silicon, aluminum, carbon and potassium elements that were generated from the results of the EDX analysis of geopolymer cross sections before and after exposure to sulfuric acid.

Nevertheless, the increase in the silicon content in the geopolymer mixtures, according to the procedure described in this paper (see Section 2.3), also has its limits from a practical point of view. With the increasing of the silica fume content, the strength evolution of the geopolymer was delayed (see Table 4) and the compressive strength of GP9.0 after 28 days of curing was significantly lower than the compressive strength of geopolymers without (GP0) or with lower amounts of silica fume (GP6.0 and GP7.5).

For the synthesis of geopolymers, the optimal range of the SiO_2_/M_2_O modulus (M = K or Na) of the alkaline silicate solution is between 1.0 and 1.5 [82,83]. By adding reactive silica fume to the solution, the SiO_2_/K_2_O modulus will rise, as certain amounts of the pozzolan will dissolve and interact with the solid content of the potassium silicate solution. As a consequence, the pH of the solution will decrease [84] and the proportion of OH^−^ in the solution may become too low for a sufficient geopolymerization [85]. Another aspect is the coordination of the silicon atoms Q^n^ resp. the number of bonds to neighboring silicon atoms in the solution. Silicon species in solution can exist as monomers (Q^0^), dimers (Q^1^) and oligomers (Q^2^, Q^3^, Q^4^), which are connected by oxygen-bridging bonds [82,86]. As the modulus of the solution increases, the number of oligomers increases sharply [82,87]. Dissolved aluminum species from the powder precursor preferably bond with Q^0^ and Q^1^ [88], which leads to an acceleration of the reaction kinetics if sufficient proportions of Q^0^ and Q^1^ are available, resp. a delayed reaction in the case of higher proportions of larger oligomers [89].

The higher strength of GP6.0 and GP7.5 compared to GP0 after 28 days can be explained by the Si/Al ratios of the geopolymers, as higher ratios comprise higher proportions of Si–O–Si bonds, which are stronger than Si–O–Al bonds [90] and, therefore, lead to higher strength [82,91]. Due to the aforementioned retarding influence of an increasing SiO_2_/K_2_O modulus of the alkaline silicate solution and the higher strength of Si–O–Si bonds, the composition and very low strength of GP9.0 suggest that a further increase in strength is to be expected in the long term due to a kinetically controlled (retardation) process. However, the literature also reports a strength loss for overly high Si/Al ratios, due to higher proportions of non-reacted particles and inhomogeneities in the network [82,92]. Thus, to further increase the silicon content in the geopolymer without the negative effect of a low degree of reaction, a longer curing period before the exposure to sulfuric acid would be favorable. Another option would be the variation of the mixing procedure of the different precursors if a gas evolution of the powdery silicon precursor should be better excluded.

The low strength resp. degree of reaction of GP9.0 after 28 days of curing was also reflected in the MIP results (see Figure 6B). Regardless of the differences of total porosity and pore size distribution of the four geopolymer mixtures, the relatively high porosity of geopolymers led to a deeper penetration of acids inside of the geopolymer and, as a consequence, to the dealumination of these areas [47]. Even with a higher silicon content, this diffusion-controlled interaction between the acid and the pore solution and the solid matrix of the geopolymer could not be prevented completely.

A major advantage of a higher silicon content in the geopolymer is the resistance of the geopolymer to surface erosion. Up to 6% silica fume, the outer edges of the specimens eroded almost immediately after the beginning of the exposure. By increasing the silica fume to 7.5% resp. 9.0%, no surface erosion could be detected.

Apart from the surface cracks (see Figure 10), the almost-intact surface of GP7.5 and GP9.0 might be explained by the statement of Sturm et al. [40], in reference to the structure of zeolite A, which consists only of Q^4^(Al)–Si species. The hydrolysis of the Si–O–Al bonds destroys the complete structure of zeolite A, as each aluminum atom is crosslinked to a silicon atom and no oxygen-bridging bonds between silicon atoms exist. For a geopolymer with a higher Si/Al ratio, which is very likely in the case of a higher silica fume content, the dealumination of the gel will also partially destroy the gel structure. However, if a sufficient number of Si–O–Si bonds remains within the structure, a complete deterioration might be prevented. The partially destroyed structure will have a higher porosity and cracks can form on the surface, but may retain an approximately intact surface, as can be seen in Figure 10. In the literature, the formation of cracks is explained by the shrinkage of the more porous deteriorated surface layer after acid exposure [27,46]. Those cracks can spread in the core of the specimens and accelerate the deterioration of the structure [46]. Bouguermouh et al. [22] reported cracks the drying of the specimen. However, in most studies, it was not specified whether cracks occur before or after drying [23,46].

The varying degrees of cracking will also affect the leaching of the elements from the geopolymer. As all geopolymer samples in this study were stored in one single container of sulfuric acid, to prevent a deviating pH caused by adding a 50% concentrated sulfuric acid solution manually in order to keep the pH constant over the whole period of exposure, no further conclusion could be made about the influence of cracks on the intensity of leaching or the progress of corrosion. Nevertheless, the differences of potassium, silicon and aluminum concentration in the sulfuric acid (see Figure 9) clearly show that potassium and aluminum were leached out easily and their concentration in the sulfuric acid solution increased with the time of exposure, whereas the silicon concentration stayed more-or-less constant and reached significantly lower concentrations. Thus, in addition to the EDX results, it can also be demonstrated by the concentration of the elements in the sulfuric acid that the corrosion mechanism was significantly characterized by the leaching of potassium and the dealumination of the geopolymer, whereas the loss of silicon from the geopolymer had only a minor influence.

The deterioration of the geopolymer also becomes visible by comparing the FTIR (see Figure 11) and XRD (see Figure 12) spectra of unexposed samples and those of the corroded layer. In the case of the XRD spectra, the shift of the broad hump indicates the dealumination of the aluminosilicate network. A similar effect can be observed by the shift of the main band in the FTIR spectra. However, for both analyses, no significant differences in the intensities of the shifts can be observed for different times of exposure.

In contrast, the EDX depth profiles reveal a change in the corroded layer over the course of exposure, namely the densification of the aforementioned area due to the increase in the silicon mole fractions. When comparing the carbon and silicon depth profiles, their direct correlation becomes obvious, as an increase in silicon after longer durations of sulfuric acid exposure automatically led to lower carbon mole fractions in the corroded layer. This was due to the silicon-induced densification of the corroded layer, which reduced the proportion of (smaller) pores and cracks in the specimen cross section and thereby also reduced the carbon mole fraction, as the pores and cracks were filled with epoxy resin, which mainly comprised carbon. The corroded layer of the geopolymer specimen with the highest amount of silica fume (GP9.0) contained the lowest carbon mole fraction compared to the other geopolymers after only 7 days of exposure, especially in the outermost area of the corroded specimen. After 84 days of exposure, GP9.0 also showed the highest degree of densification, as the silicon mole fraction in the corroded layer reached approximately 27%. For the potassium and aluminum mole fractions in the corroded layer, no significant changes over the time of exposure could be observed, leading to the assumption that the leaching of potassium and the dealumination of the network were not subject to any significant change over time.

It has already been mentioned that the EDX results have a semi-quantitative character. To improve the quality of the EDX results, all elements in the specimen cross section were considered (see Section 2.7). Furthermore, the manual corrections of the heights of the 100 µm segments could ensure that the molar composition of each segment of the specimen’s cross section was not distorted by the high mole fractions of the carbon, which occurred as a result of epoxy resin-filled air voids or the presence of larger cracks (see Figure 3). To be able to assess the actual quality of the EDX measurements resp. the depth profiles of the elements, Table 6 shows the mean element mole fractions of the geopolymers after 28 days of curing before exposure to sulfuric acid, determined by EDX elemental mappings. To compare the values to the mole fractions in Table 3, namely the calculated mole fractions of the elements in the geopolymer composition, the EDX determined mole fraction of each element was normalized to the new sum of all elements minus the carbon.

**Table 6:** Mean mole fraction of elements in the geopolymer pastes (EDX results from geopolymer specimens after 28 days of curing, before exposure to sulfuric acid).

Element	GP0	GP6.0	GP7.5	GP9.0
Si	21.44	21.69	22.55	22.88
Al	10.63	9.74	9.86	9.75
Fe	0.81	0.67	0.76	0.67
Ca	0.77	0.65	0.72	0.65
Ti	0.18	0.22	0.17	0.18
Na	0.20	0.13	0.10	0.11
K	5.00	4.93	4.97	4.96
Mg	0.19	0.14	0.20	0.18
O	60.78	61.83	60.68	60.61
Total	100	100	100	100

**Figure 17:**
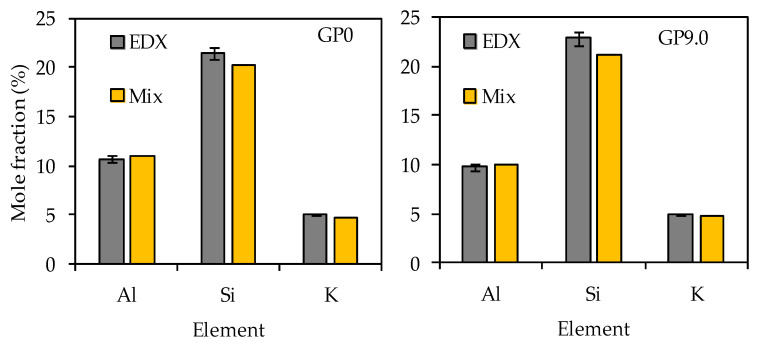
Silicon, aluminum and potassium mole fractions of geopolymers GP0 and GP9.0 before exposure to sulfuric acid. Calculated mole fractions of geopolymer composition (Mix) and mole fractions determined by EDX elemental mappings (EDX).

The comparison of the calculated mole fractions (see Table 3) and the EDX mole fractions (see Table 6) reveal that, especially for the most relevant elements, namely silicon, aluminum and potassium, the deviations of the values were small (see Figure 17).

For all four geopolymers, the EDX mole fractions of aluminum and potassium were always slightly higher than the calculated mole fractions of the geopolymer composition, as shown in Figure 17, exemplified by GP0 and GP9.0. The opposite is true for the silicon mole fractions. The comparison of the calculated mole fractions with the mole fractions determined by EDX elemental mappings reveal that, despite the semi-quantitative nature of the EDX elemental mappings (for degradated samples), good quantitative results were achieved on the reference (non-degradated) samples.

## 5. Conclusions

Specific depth profiles on the basis of the proposed EDX elemental mappings of geopolymers after sulfuric acid exposure are a useful tool to detect the change of elemental composition in the corroded layer, and the progress of corrosion, when all present elements are taken into account and larger air voids and cracks are excluded. In this context, the following conclusions can be drawn:

1. The substitution of 7.5% and 9.0% of metakaolin by silica fume leads to the formation of silicate-rich geopolymer structures, which are resistant against surface erosion due to dissolution reactions in sulfuric acid;
2. For all of the silica fume dosages, a densification of large areas of the corroded layer of specimens can be observed due to the formation of new silicon-rich gels in the aforementioned area;
3. The post-densification of large areas of the corroded layer is more pronounced and sets in at earlier exposure durations if the amount of silica fume increases, which decelerates the progress of corrosion;
4. Higher proportions of silica fume have a negative influence on strength evolution of geopolymer pastes;
5. The strength and durability of geopolymers could be further increased if the negative effect of higher amounts of silica fume on the geopolymerization retardation could be prevented.

## Figures and Tables

**Table 1:** Chemical composition (wt%) of metakaolin and silica fume.

Oxides	Metakaolin (wt%)	Silica Fume (wt%)
SiO_2_	58.80	97.02
Al_2_O_3_	32.79	0.55
Fe_2_O_3_	3.50	0.21
CaO	2.35	0.32
TiO_2_	1.80	0.00
Na_2_O	0.06	0.18
K_2_O	0.35	0.98
MgO	0.17	0.49

**Table 2:** Mass fraction of metakaolin, silica fume and potassium silicate solution and l/s-ratio of the geopolymer pastes GP0, GP6.0, GP7.5 and GP9.0.

Geopolymer	Metakaolin (%)	Silica Fume (%)	Potassium Silicate Solution (%)	l/s (-)
GP0	64.81	0.00	35.19	0.54
GP6.0	60.92	3.89	35.19	0.54
GP7.5	59.95	4.86	35.19	0.54
GP9.0	58.98	5.83	35.19	0.54

**Table 3:** Mole fraction of elements in the geopolymer pastes.

Element	GP0	GP6.0	GP7.5	GP9.0
Si	20.22	20.85	21.01	21.17
Al	11.05	10.38	10.22	10.05
Fe	0.75	0.71	0.70	0.69
Ca	0.72	0.68	0.67	0.66
Ti	0.39	0.36	0.36	0.35
Na	0.03	0.04	0.04	0.04
K	4.68	4.69	4.69	4.69
Mg	0.07	0.08	0.08	0.08
O	62.08	62.20	62.23	62.26
Total	100	100	100	100

**Table 4:** Compressive strength of geopolymers after 1, 7 and 28 days of curing (1d, 7d, 28d).

Geopolymer	Compressive Strength (MPa)		
	1d	7d	28d
GP0	60.8	53.6	58.0
GP6.0	13.9	65.7	81.3
GP7.5	-	15.4	71.9
GP9.0	-	-	10.7

**Table 5:** Depth of erosion of geopolymers after different durations of exposure.

Geopolymer	Depth of Erosion (mm)				
	7d	14d	28d	56d	84d
GP0	1.04	1.01	1.14	1.16	1.07
GP6.0	0.89	0.78	0.96	0.83	0.94
GP7.5	0.00	0.00	0.00	0.00	0.00
GP9.0	0.00	0.00	0.00	0.00	0.00

## Data Availability

Not applicable.
